# Correlates of serum lipoprotein (A) in children and adolescents in the United States. The third National Health Nutrition and Examination Survey (NHANES-III)

**DOI:** 10.1186/1476-511X-3-29

**Published:** 2004-12-16

**Authors:** Thomas O Obisesan, Muktar H Aliyu, Abayomi S Adediran, Vernon Bond, Celia J Maxwell, Charles N Rotimi

**Affiliations:** 1 Section of Geriatrics, Department of Medicine, Howard University Hospital, Washington, USA; 2 Department of Epidemiology, University of Alabama at Birmingham, USA; 3 Department of Human Health & Leisure Studies, Howard University, Washington, USA; 4 Institute for Women's Health, Howard University Hospital, Washington, USA; 5 National Human Genome Center Genetic Epidemiology Unit, Department of Microbiology, Howard University, Washington, USA

**Keywords:** Lipoprotein(a), Adolescence, Gender, Ethnicity, Parental History

## Abstract

**Objective:**

To determine the correlates of serum lipoprotein (a) (Lp(a)) in children and adolescents in the United States.

**Methods:**

Cross-sectional study using representative data from a US national sample for persons aged 4–19 years participating in The Third National Health Nutrition and Examination Survey (NHANES-III).

**Results:**

We observed ethnicity-related differences in levels of Lp(a) > 30 mg/dl, with values being markedly higher in African American (black) than nonhispanic white (white) and Mexican American children in multivariate model (*P *< 0.001). Higher levels of Lp(a) > 30 mg/dl associated with parental history of body mass index and residence in metro compared to nonmetro in Blacks, and high birth weight in Mexican American children in the NHANES-III. In the entire group, total cholesterol (which included Lp(a)) and parental history of premature heart attack/angina before age 50 (*P *< 0.02) showed consistent, independent, positive association with Lp(a). In subgroup analysis, this association was only evident in white (*P *= 0.04) and black (*P *= 0.05) children. However, no such collective consistent associations of Lp(a) were found with age, gender, or birth weight.

**Conclusion:**

Ethnicity-related differences in mean Lp(a) exist among children and adolescents in the United States and parental history of premature heart attack/angina significantly associated with levels of Lp(a) in children. Further research on the associations of Lp(a) levels in childhood with subsequent risk of atherosclerosis is needed.

## Introduction

Levels of serum or plasma Lp(a) above 30 mg/dL are associated with increased risk of coronary artery disease and stroke in adults of European descent [1-3]. Given the high degree of structural homology of one of the domains of its apolipoprotein(a) component with plasminogen, one proposed mechanism is interference with thrombolysis [1,3]. Adults of African descent have mean levels of Lp(a) approximately twice those of Europeans but do not have commensurately increased risk of atherosclerotic disease; nor has Lp(a) been shown to be a coronary artery disease risk factor in Blacks [4,5]. The explanations for the differential effects of Lp(a) on CVD risk among different populations are poorly understood. Because birth weight has been shown to influence levels of Lp(a) [6], and adverse patterns of blood lipids and atherosclerosis itself begin in childhood, studies of population and individual differences in the early onset and progression of risk factors through adolescence are important [7]. Given the reported contribution of intrinsic factors, family history, and environmental factors to the CVD risk in adults [8-10], the identification of inherited risk markers and environmental variables that may interact with levels of Lp(a) > 30 mg/dl to modify its influence on the development of atherosclerosis at an early age, is therefore imperative.

Few studies have examined the epidemiology of Lp(a) in representative samples of total populations of children and adolescents[11,12]. However, no study has examined whether the effects of inherited and acquired or environmental factors interact with Lp(a) > 30 mg/dl, to cause differential attributable risk in different populations using data from a nationally representative sample of children in the US. We utilized data from a national survey of over 30,000 persons age 1 year and older with extensive blood lipid data to examine correlates of Lp(a) in children and adolescents and specifically to determine whether: [1] ethnic differences in shape of Lp(a) distributions seen in adults are also seen as early as age 4 in children; [2] family history of cardiovascular disease is associated with higher levels of Lp(a); [3] the effects of ethnicity and family history of CVD on the levels of Lp(a) are influenced by low birth weight, other personal, behavioral or environmental variables.

## Methods

Data for this analysis was obtained from The Third National Health and Nutrition Examination Survey (NHANES-III) conducted on a nationwide multi-stage probability sample of about 40,000 persons from the civilian, non-institutionalized population aged 2 months and over of the United States excluding reservation lands of American Indians. Of these, 31,311 were examined. Our analysis was restricted to children aged 4–11 years (518 whites, 877 blacks, and 685 Mexican Americans) and adolescents aged 4–19 years (336 Whites, 665 Blacks and 504 Mexican Americans) with valid Lp(a) measurements in Phase II of the survey conducted in 1991–1994. Details of the planning, sampling, operation, informed consent procedures, and measures taken to maintain confidentiality of information have been previously detailed [13].

Demographic, medical history and behavioral information were collected prior to the examination by household interview of the parents or guardians of children and of adolescents aged 12 and over. Parents of children aged 2 months-11 years were asked "How much did the child weigh at birth?". Parents responding "don't know" were asked "Did the child weigh more than 5 1/2 pounds (2500 grams) or less? Responders were then asked, "Did the child weigh more than 9 pounds (4100 grams) or less?" An approximate category of weight at birth was created by combined responses to exact birth weights and the latter two questions. Participants' parent or guardian was also asked, "Has either of the biological parents ever been told by a doctor that he or she had a) high blood pressure or stroke before age 50 b) heart attack or angina before the age of 50? c) high blood cholesterol at any age? d) diabetes at any age? All "Yes" responses were followed by "Which, father, mother, or both?" Other interview variables are described elsewhere [13].

Blood samples were obtained at the examination centers [14]. A subsample of persons 12 years and over was asked to fast overnight for the examination of lipids in the morning. Lp(a) in serum was measured immunochemically by using an enzyme-lined immunosorbant assay (ELISA) (Strategic Diagnostics, Newark, DE) [14], which does not have cross reactivity with plasminogen or LDL non sensitive to apo(a) size heterogeneity. The normal range was set at 0 to 30 mg/dL because concentrations above 30 mg/dL have been associated with increase risk for coronary heart disease and stroke [1,3] (Plasma concentrations were 3 % lower than serum concentrations). The quality control of the Lp(a) assay has been described in detail elsewhere [14]. Serum samples with Lp(a) > 80 mg/dL were diluted into the assay range with sample diluent.

Serum total cholesterol were determined at the Centers for Disease Control using a modified ferric chloride technique (GFAA/Perkin-Elmer Model 3030 and 5100) [14]. High-density lipoprotein (HDL) was measured in serum following the precipitation of other lipoproteins with a polyanion/divalent cation mixture and triglycerides were measured enzymatically by Hitachi 704 Analyzer autoanalyzer (Boehringer-Mannheim Diagnostics). LDL cholesterol level was calculated by the Friedewald equation for individuals 12 years and older who were examined in the morning and fasted 9 hours or more, and whose triglyceride concentration was less than or equal to 400 mg/dL. Because fasting was not required in children, LDL could be calculated on only 15% of this sample. In selected analyses, serum total cholesterol was corrected for Lp(a) cholesterol as follows: TCc = TC - Lp(a) × 0.30 [11]. Standing height was measured to the nearest 0.1 centimeter, weight to the nearest 0.01 kg, triceps, subscapular, suprailiac and mid-thigh skinfold thickness to the nearest 0.1 millimeter and waist and buttocks circumference to the nearest 0.1 centimeter [15,16].

### Statistical analysis

Population estimates for many of the variables other than Lp(a) have been published by the National Center for Health Statistics [14,17]. Because, body weight, family history, socio-economic factors including income, gender, ethnicity, birth weight and regional diversity have been shown to influence levels of Lp(a) and or CVD risk in general, [18,6,21] our analyses of the population estimate and correlates of Lp(a) were mindful of these factors. In order to ensure adequate weight for a given age group, and to examine pre- peri- and post pubertal effects on levels of Lp(a), quintile distribution of age was used as a categorical variable. Detailed descriptive statistics and measures of association were computed initially using unweighted data. Kendall's nonparametric rank correlation was used to assess the association of Lp(a) with other variables and compared to Pearson correlation [22]. To determine the influence of gender and ethnicity on the distribution of Lp(a), analysis of covariance was used to compute adjusted means for subjects within sex and ethnic categories, and to assess the statistical significance of differences of means among groups. Stepwise logistic multiple regression analysis was used to develop models for predicting Lp(a) >30 mg/dL for each sex, and ethnic group [22]. Only variables with pre-specified hypotheses and with statistically significant univariate correlation coefficients were eligible to enter the regression models. Following these preliminary analyses, preplanned hypotheses and major findings of the unweighted analyses were confirmed using techniques that incorporated sampling weights and design features of the survey [14]. Population estimates for mean Lp(a) and percentiles and statistical tests of weighted proportions were produced using Statistical Analysis System (SAS) callable SUDAAN [23]. Chi-Square analysis was used for the comparisons of distributions of Lp(a) categorized into 10 mg/dL strata between sex, ethnicity and age groups. Associations of Lp(a) with other variables were confirmed in final weighted analysis, using PROC LOGIST procedure in SUDAAN [23] with alpha set at *<*0.05. Since substantial proportions of white and Mexican American children had undetectable Lp(a), log or other transformations could not produce an approximately normal distribution of Lp(a) for parametric analyses. Therefore analytic results presented are primarily those using Lp(a) > 30 mg/dL as a categorized variable.

## Results

### Univariate Analyses

#### Ethnicity

**Figure 1:**
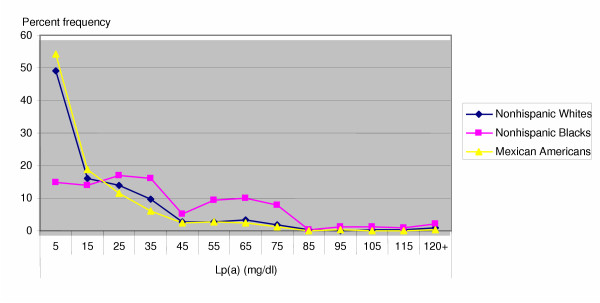
Percent Frequency of Lp(a) mg/dl in children aged 4–16 years, by ethnic group in the Third National Health and Nutrition Examination Survey, 1988–1994.

Blacks had higher median Lp(a) than whites, who had higher levels than Mexican Americans (Table 1). The difference was already apparent at ages 4–5 years. Further, the shape of the Lp(a) distributions differed markedly for BLACKS compared to other ethnic groups at each age and overall. Blacks had a bimodal distribution that was less skewed than whites or Mexican Americans (Figure 1). The percentage of children aged 4–19 with Lp(a) > 30 mg/dL, was higher in Blacks (54.3, SE 1.8) than in Whites (20.3, SE 2.4) or Mexican Americans (16.8, SE 2.3), both overall (Chi square 47.4, p < 0.001) and in each age group (Table 1).

**Table 1:** Selected percentiles of lipoprotein(a) distributions and prevalence of concentrations > 30 mg/dL in children and young adults aged 4–19 years by ethnic group and age: NHANES-III, 1988–1994.

Ethnic group	Age (yrs)	Lipoprotein(a) (mg/dL)						N
		Percentile					Percent > 30 (mg/dL)	
		5	10	50	90	95		
Nonhispanic white								
	4–5	0	0	7	38	62	15.0	214
	6–11	0	0	12	48	65	18.8	304
	12–15	0	0	10	48	56	20.2	187
	16–19	0	0	9	53	62	25.8	149
Nonhispanic black								
	4–5	2	6	31	75	94	52.5*	303
	6–11	1	5	32	76	100	53.3*	574
	12–15	0	5	33	77	95	56.4*	358
	16–19	1	6	31	69	76	54.6*	307
Mexican American								
	4–5	0	0	5	30	48	8.2	309
	6–11	0	0	9	45	62	21.0	376
	12–15	0	0	9	48	58	20.1	272
	16–19	0	0	8	36	52	11.4	232

* Indicates unadjusted statistically significant difference in levels of Lp(a) > 30 mg/dl between black and white children, and between black and Mexican American children in the same age group. Significance level was set at < 0.05.

#### Age

Table 3 shows percentiles by sex, age and ethnic group. Among boys of all sex-ethnicity groups, median Lp(a) was higher at age 6–11 than at age 4–5, then tended to decline slightly through age 16–19. Among girls, there was no consistent pattern for median Lp(a), being highest at age 16–19 in whites, at age 12–15 in blacks, and at 6–11 in Mexican Americans. The percentage with Lp(a) > 30 mg/dL varied significantly by age group only in Mexican Americans (Chi square = 10.6, *P *= 0.02) (Table 1).

**Table 3:** Selected percentiles of lipoprotein(a) distributions and prevalence of concentrations > 30 mg/dL in children and young adults aged 4–19 years by ethnic group and age: NHANES-III, 1988–1994.

Ethnicity	Age (Yrs)	Lipoprotein(a) mg/dL					N
		Percentile					
		5	10	50	90	95	
		Girls					
Nonhispanic white							
	4–5	0	0	7	35	62	113
	6–11	0	0	9.5	34	60	146
	12–15	0	0	8.5	47	55	106
	16–19	0	0	13	54	61	84
Nonhispanic black							
	4–5	1	7	32.5	74	100	146
	6–11	0	3	31	72	98	288
	12–15	2	9	34	78	102	191
	16–19	1	5	30	71	80	166
Mexican American							
	4–5	0	0	6	28	36	152
	6–11	0	0	11	46	59	177
	12–15	0	0	9	47	56.5	140
	16–19	0	0	10	34	52	119
		Boys					
Nonhispanic white							
	4–5	0	0	7	38	62	101
	6–11	0	0	17	63	75	158
	12–15	0	0	11	49	56	81
	16–19	0	0	7	38	65	65
Nonhispanic black							
	4–5	2	5	31	75	94	157
	6–11	2	6	34	78	105	286
	12–15	0	2	32	75	78	167
	16–19	0	9	32	66	75	141
Mexican American							
	4–5	0	0	5	34	57	157
	6–11	0	0	8	45	67	199
	12–15	0	0	8.5	49	60	132
	16–19	0	0	7	37	55	113

#### Gender

Median Lp(a) did not differ consistently by gender across age or ethnic groups (Table 3). Similarly, the percentage with Lp(a) > 30 mg/dL did not differ significantly by gender within ethnic groups (Chi Square = 0.003, *P *= 0.96).

#### Birth Weight

Birth weight by parental recall was available for children aged 4–11 years (Table 4). Birth weight did not vary with Lp(a) in any ethnic group. Further, in blacks, the percentage with Lp(a) >30 mg/dL did not differ by birth weight category (52.5, 53.3, 56.4, respectively). Small numbers of cases with both abnormal birth weight and elevated Lp(a) >30 mg/dL among whites and Mexican Americans precluded meaningful analysis.

**Table 4:** Selected percentiles of lipoprotein(a) distributions and prevalence of concentrations > 30 mg/dL in children aged 4–11 years by ethnic group and birth-weight: NHANES-III, 1988–1994.

Ethnic group	Age (yrs)	Lipoprotein(a) mg/dL		N
	Birth Weight	Median 50	Percent > 30 mg/dL	
Nonhispanic white				
	<2500 gm	12	15.0	32
	2500–4100 gm	10	18.8	441
	>4100 gm	7	20.2	43
Nonhispanic black				
	<2500 gm	32	52.5	115
	2500–4100 gm	32	53.3	702
	>4100 gm	29	56.4	44
Mexican American				
	<2500 gm	3	8.2	45
	2500–4100 gm	8	21.0*	573
	>4100 gm	5	20.1*	57

* Indicates within group statistically significant association of Lp(a) > 30 mg/dl with birth weight using birth weight < 2500 gm as the reference value. Significance level was set at < 0.05.

#### Family history

**Figure 2:**
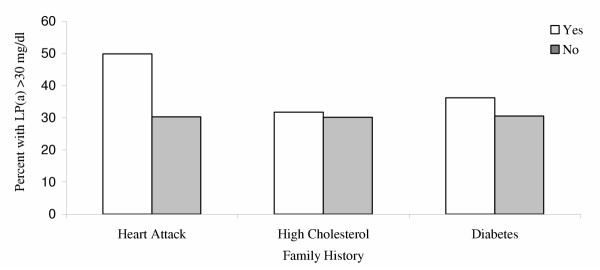
Prevalence of lipoprotein(a) concentration > 30 mg/dl in children aged 4–16 years, by combined ethnic group, and parental history of heart disease or angina, high cholesterol, or diabetes below age 50 in the Third National Health and Nutrition Examination Survey, 1988–1994

In all the groups combined (age range 4–16 years), the percentage with Lp(a) > 30 mg/dL was significantly higher among those with parental history of heart attack/angina before age 50 years compared to those without (50.0 percent versus 30.2 percent), Chi square 2.72, *P *= 0.011), whereas the percentage (Lp(a) > 30 mg/dL) was similar among those with parental history of diabetes and high cholesterol vs. those without (Figure 2). Due to small numbers of children with a history of heart attack within ethnic groups, the difference in percentage of persons with Lp(a) did not attain significance in within-groups analysis: white children, 42.56% versus 18.36%, *P *= 0.23, black children 72.37% versus 54.06%, *P *= 0.12, Mexican American children 34.46% versus 18.26%, *P *= 0.33 (Table 2). In white children, the percentage was higher in children with a parent with high blood cholesterol compared to children without (26.74% versus 16.12%; *P *= 0.02). However, no significant differences were seen in other groups.

**Table 2:** Median lipoprotein(a) and prevalence of concentrations >30 mg/dL in children aged 4–16 years by ethnic group and parental history of heart or an angina below age 50, high cholesterol, or diabetes: NHANES-III, 1988–1994

Ethnic group		Lipoprotein(a) mg/dL						N		
		Median (mg/dL)			Percent > 30 (mg/dL)					
		Heart Attack	High Cholesterol	Diabetes	Heart Attack	High Cholesterol	Diabetes	Heart Attack	High Cholesterol	Diabetes
Nonhispanic white										
	Yes	25	13	12.5	42.6*	26.7	32.8	15	138.0	34
	No	10	8	8	18.4	16.1	18.0	722	586.0	701
Nonhispanic black										
	Yes	45	28	33.5	72.4*	48.9	56.6	53	111.0	86
	No	32	33	32	54.1	55.5	54.8	1242	1176.0	1206
Mexican American										
	Yes	12	9.5	7	34.5*	19.6	19.2	21	134.0	45
	No	7.5	7	8	18.3	18.7	18.7	990	870.0	966

* Indicates statistically significant association of parental history of heart attack before age 50 with levels of Lp(a) > 30 mg/dl nonhispanic black, nonhispanic white and Mexican American children. Significance level was set at < 0.05.

#### Region

Among white children, median Lp(a) was lower in the Midwest (8.2 mg/dL) and South (8.2) than in the Northeast (16.4), or West (10.1). No differences were noted for Blacks and too few Mexican Americans lived in the Northeast and Midwest for evaluation. In white children, the percentage with Lp(a) > 30 mg/dL were 15.1 in the Midwest, Northeast 25.1, South 21.2, West 21.4 (*P *= 0.56). Among blacks, a greater percent of metropolitan, compared to non-metropolitan (57% Vs 49.7%; P = 0.04) had Lp(a) > 30 mg/dL. No significant differences were seen in other groups.

#### Income

Family income < $20,000 was not associated with Lp(a) > 30 mg/dL in whites or Mexicans, but Blacks with low income tended to have a higher percentage of individuals with Lp(a) >30 mg/dL (56.9% versus 50.5%, *P *= 0.10). Poverty income ratio was not significantly correlated with Lp(a).

#### Multivariate Analyses

The following additional variables known to influence CVD risk were assessed as correlates of Lp(a) by ethnic group: age, total serum cholesterol, HDL cholesterol, casual triglycerides, hours of fasting, weight, height, body mass index, waist circumference, waist-to-hip ratio, subscapular skinfold thickness, suprailiac skinfold thickness, pulse rate, systolic and diastolic blood pressure, heavy activity frequency or TV hours. Logistic regression analysis with Lp(a) > 30 as dichotomous dependent variable and age in months as independent variable revealed a significant linear association in whites (beta = 0.004, SE = 0.002, *P *= 0.03) and a quadratic association in Mexican Americans (age beta 0.068, SE = 0.013, *P *< 0.001, age squared beta -0.000, SE beta 0.000, *P *< 0.001), indicating a lower prevalence of Lp(a) > 30 mg/dL at both ages 4–5 and 16–19 than at 6–15 years. Age and age squared, and sex were entered first in all analyses described below. Controlling for age, sex was not significantly associated with high Lp(a) in any group. Compared to whites, non-hispanic black ethnicity was significantly associated with high Lp(a) after controlling for age and sex (*P *< 0.001). Mexican American ethnicity was not significantly associated with lower prevalence of high Lp(a) (*P *= 0.35). Within ethnic groups at ages 4–11 years, low birth weight (<2500 g) was not significantly associated with high Lp(a) after controlling for age and sex. High birth weight (>4100 g) was associated with high Lp(a) (beta 1.87, *P *= 0.02) only in Mexican Americans.

Parental history of heart attack/angina before age 50 was significantly associated with Lp(a) >30 after controlling for age and sex both in white children (beta -1.14, SE 0.55, *P *= 0.04) and in blacks (beta -0.80, SE 0.39, *P *= 0.05). Parental history of heart attack/angina was also significantly associated with high Lp(a) in all children (*P *= 0.02) after controlling for age, sex and ethnicity. In white children, parental history of high blood cholesterol (*P *= 0.07) and diabetes mellitus (*P *= 0.16) were not significantly associated with high Lp(a) after adjustment for age and sex.

Residence in central cities/fringe areas remained significantly associated with high Lp(a) in black children after controlling for age, sex, region, season, and time of the day (beta = -0.33, SE = 0.13, *P *= 0.02). Region, rural/urban code, family income < 20,000 or higher poverty income ratio were not significantly associated with high Lp(a) in white or Mexican American children (all *P *> 0.05).

Body mass index or weight were significant predictors of Lp(a) > 30 mg/dL only among black children after controlling for age, sex, region, rural/urban code, season and poverty income ratio, e.g. weight(kg) *P *= 0.02. Neither HDL cholesterol nor casual triglyceride concentration was significantly associated with Lp(a) after controlling for multiple variables. Total serum cholesterol was significantly associated with Lp(a) after controlling for multiple variables in all three ethnic groups as expected.

## Discussion

The most important findings of this study are that ethnicity significantly associated with Lp(a), and that parental history of heart attack significantly associated with Lp(a) levels >= 30 mg/dl. While non-hispanic black children had significantly higher total Lp(a) level, compared to white and Mexican American children, no consistent associations of age or gender with Lp(a) were found in NHANES-III below age 20. Low birth weight (<2500 g) was not significantly associated with high Lp(a) after controlling for age and sex in the entire group. Higher levels of Lp(a) >30 mg/dl was evident in metropolitan compared to non-metropolitan non-hispanic black children.

### Mechanisms

Lp(a) is a circulating particle that consists of phospholipids, cholesterol, and apolipoprotein B-100 (i.e. a LDL particle), with apolipoprotein(a) attached to the latter at a single point [5,24]. Like LDL, Lp(a) when oxidized may promote atherosclerosis by promoting formation of foam cells which release growth factors. Lp(a) acquires a pathogenic profile on entering the arterial cell wall as a result of the influence of factors operating in the inflammatory environment of the atheromatous vessel, such as proteolytic enzymes of the metalloproteinase family [25]. About 80% of the amino acids in apo(a) are homologous with those of plasminogen, suggesting a possibly thrombolytic effect which might both promote atherosclerosis and trigger acute thrombotic occlusions [1,3,26].

Whereas levels of Lp(a) above 30 mg/dL was shown to increase risk of coronary heart disease in European samples [3,24], no such association has been found in black populations, in whom the concentration is twice that in Europeans [24,27]. Study of serum concentrations of this particle in children is especially important since, unlike LDL, its concentration is postulated to be remarkably stable throughout the life of an individual. Thus, identification of persons at increased risk early in life would permit more effective intervention to lower levels of modifiable risk factors such as LDL cholesterol.

### Environment, Ethnicity, Age and Gender

A number of studies of adults have compared Lp(a) levels in Whites and Blacks, and have uniformly reported two-fold higher levels in Blacks [4,5,11]. In Texas, Mexican American adults were found to have lower Lp(a) than whites. Conversely, Kambor et al observed higher mean and median plasma Lp(a) concentrations in hispanic men than white men in Colorado with lesser difference seen in women[28]. Although the explanations for these findings remain unclear, environmental factors, genetic admixture [29] or a combination of both should be considered.

No previous reports of studies comparing Lp(a) in whites and blacks and Mexican American children in the same study were found prior to NHANES-III. In fact, few comparisons of plasma Lp(a) concentrations were found for hispanic children or other children below age 8 years prior to NHANES-III [30,31]. The present findings extend the published report by examining children in greater detail and examining the relationship of birth weight and family history of cardiovascular disease with Lp(a) in children.

Perhaps one of the most noteworthy observation from this study is the significantly higher levels of Lp(a) >30 mg/dl in black, compared to white and Mexican American children (Table 1). Ethnic-related differences in Lp(a) similar to those in adults were found in children as young as 4–5 years of age, supporting the presence of higher levels of Lp(a) in black children compared to other ethnic groups (Table 1). This observation is consistent with findings of the Bogalusa Heart Study of white and black children, and the NHLBI Growth and Health Study of girls that showed higher Lp(a) levels in black than white children [30,32]. Findings in Mexican Americans in the NHANES-III study is analogous to reports from the Colorado study showing a greater percent of Hispanics than whites (19% versus 12%) to have Lp(a) > 25 mg/dL.

The explanation for ethnic-related differences in levels of Lp(a) in the US remains unclear. Other than total cholesterol, no single environmental or biological variable consistently associated with levels of Lp(a) in the NHANES-III sample. While levels of Lp(a) > 30 mg/dl received significant contributions from BMI and residence in metro compared to non-metro in black children, only higher birth weight significantly contributed to levels of Lp(a) in Mexican American children. Contrary to a previous report of association of low birth weight with elevated Lp(a) concentration in black children, [6] we found no consistent association of low birth weight with levels of Lp(a) in black or white children in the present study (Table 4).

At the genetic level, heritability estimates were reportedly higher for Whites than for Blacks [33,34] despite the disproportionately higher levels of Lp(a) in Blacks. This observation raises an important question about the genetic determinants of differential levels of Lp(a) in non-hispanic Blacks compared to Whites. A recent study on genetic linkage analysis by Barkley et al found no linkage evidence to support the presence of a single but separate gene with large effects specifically segregating in non-hispanic Blacks that may account for elevated Lp(a) levels[35] Conversely, high levels of Lp(a) levels have been suggested to be an old African trait that is associated with mutations in the coding sequences of apo(a) [36]. Collectively, these disagreements among studies suggest that higher levels of Lp(a) in non-hispanic Blacks compared to other ethnic groups, may result from a complex interaction of genes with environmental and metabolic factors, [37]. Future identification of the presence and nature of this interaction is imperative.

Studies of adults found an association of higher age and female gender with higher Lp(a) levels [38]. The Bogalusa Heart Study found a small but significant gender difference and a weak positive correlation with age (p < 0.001) in white girls 11–17 years of age [30]. However, we found no consistent associations of age or gender with Lp(a) in NHANES-III below age 20 (Table 3). Despite the levels of Lp(a) that tended to be highest between age 6 – 11 years in boys, the lack of similar trend in girls, and the absence of age-related difference in the levels of Lp(a) in the combined group, suggests that pre-pubertal or pubertal status may not significantly influence levels of Lp(a) in children.

Altogether, our observation from the present NHANES-III study, together with the work of others [30,38] found very few significant associations of Lp(a) with personal, behavioral, or environmental variables. It therefore appears likely, that multiple factors at the environment and or genetic levels may act together to differentially influence levels of Lp(a) in children and adolescents in the US.

### Family History

Few studies of the association of Lp(a) with family history have been reported in children [30,39]. Our observations of significant association of parental history of heart attack/angina before age 50, with levels of Lp(a) >30 mg/dl in NHANES-III (Figure 2) are consistent with results from the Bogalusa study that found an association of parental history of premature heart attack with higher levels of Lp(a) [30]. However, contrary to Bogalusa study showing an association of Lp(a) with parental history of hypercholesterolemia, trends for family history in black children in NHANES-III were concordant with those in whites, although not attaining statistical significance. More recently, Dirisamer and colleagues provided additional support for higher levels of Lp(a) levels in children and adolescents from families with premature coronary heart disease compared to those without familial coronary heart disease [40]. In young adults aged 23–35 years in the CARDIA study, a non-significant trend toward higher Lp(a) levels in those with a family history of myocardial infarction was observed in whites, but no association was seen in blacks [33]. Collectively, the association of parental history of premature heart attack appears associated with levels of Lp(a) > 30 mg/dl, may lend support to the theory of genetic underpinning for the higher levels of Lp(a) observed in black children.

In the NHANES-III study, no association of Lp(a) were seen with family history of stroke, hypertension, or diabetes. Similarly, family history of high cholesterol and diabetes were not significantly associated with levels of Lp(a) >30 mg/dl in the entire sample (Figure 2), except in non-hispanic white children (Table 2). Cross-sectional studies of adults have not consistently shown a relationship of Lp(a) with NIDDM. Conversely, several reports indicate an association between IDDM with Lp(a) [29,41]. However, NIDDM is thought to have a stronger genetic component in its etiology than IDDM [42]. Despite the inconsistencies in the literature, there is strong evidence to suggest that Lp(a) is a risk factor for vascular disease in diabetics [43]. Further research on clinical and subclinical diabetes and Lp(a) is needed.

### Limitations

Limitations of the present study include possible bias from survey non-response, missing values for some variables, and confounding by variables not measured. Fortunately, several special studies of earlier NHANES-III data have indicated little bias due to non-response [44]. Although, adequate reliability has been demonstrated for Lp(a) measurement [14], the lack of a single, generally accepted laboratory method and national standardization program remains a problem, perhaps explaining in part the inconsistencies among studies [3]. The relatively large sample size provided good statistical power and the conservative criteria for statistical significance reduced the possibility of chance findings attaining significance despite a large number of tests. Overall, the representativeness of the sample and the use of sample weights provided wide generalizability of the results to United States black and white and Mexican American children and adolescents of the same ages.

In conclusion, ethnicity significantly associated with levels of Lp(a). Parental history of heart attack/angina before age 50 years associated with levels of Lp(a) > 30 mg/dl in offspring. Collectively, different pathological thresholds may have to be established for elevated serum Lp(a) levels, to be used as a risk marker for coronary heart disease in different populations. Future research should include longitudinal studies of Lp(a) in white, black and hispanic children followed to adulthood. Racial admixture as well as environment and behavioral variables associated with acculturation and urban residence should be studied, especially in Mexican American and black populations. Standardization of methods will facilitate inter-study and longitudinal comparisons.
